# Supplementary material for: Diffusion microstructure alterations in subcortical gray matter are associated with cognitive and motor performance in Parkinson’s disease: a pilot study
Source: Front Neurol. 2026 Jul 29;17:1863964. doi: 10.3389/fneur.2026.1863964 (PMC13464544; doi:10.3389/fneur.2026.1863964)
Supplement: Supplementary file 1 [file Table_1.docx]

**Supplemental Table Captions**

**Table 1:** Mean, standard deviation, and t-test p-values for each of the demographic, behavioral, and total brain volume control metrics used in the analysis stratified and compared by acquisition site.

**Table 2:** Mean and standard deviation values for each of the microstructural metrics included in this study, within each of the 14 ROIs examined, at both baseline and follow-up timepoints.

**Table 3:** Slopes, p-values, and p-values adjusted for multiple comparisons using the Benjamini and Hochberg (1995) method for the association between MOCA score and each of the microstructural metrics averaged within the nine subcortical ROIs examined in this study.

**Table 4:** Slopes, p-values, and p-values adjusted for multiple comparisons using the Benjamini and Hochberg (1995) method for the association between Executive Function Z-Score and each of the microstructural metrics averaged within the nine subcortical ROIs examined in this study.

**Table 5:** Slopes, p-values, and p-values adjusted for multiple comparisons using the Benjamini and Hochberg (1995) method for the association between Attention Z-Score and each of the microstructural metrics averaged within the nine subcortical ROIs examined in this study.

**Table 6:** Slopes, p-values, and p-values adjusted for multiple comparisons using the Benjamini and Hochberg (1995) method for the association between Language Z-Score and each of the microstructural metrics averaged within the nine subcortical ROIs examined in this study.

**Table 7:** Slopes, p-values, and p-values adjusted for multiple comparisons using the Benjamini and Hochberg (1995) method for the association between Memory-Visuospatial Z-Score and each of the microstructural metrics averaged within the nine subcortical ROIs examined in this study.

**Table 8:** Slopes, p-values, and p-values adjusted for multiple comparisons using the Benjamini and Hochberg (1995) method for the association between Latent Fitness controlled for Baseline Latent Fitness and each of the microstructural metrics averaged within the nine subcortical ROIs examined in this study.

**Table 9:** Volumetric associations between bilateral subcortical structures, calculated using freesurfer, and the behavioral metrics measured in this study. Displayed are slope (F-values) and p-values, adjusted for multiple comparisons using the Benjamini and Hochberg (1995) method.

**Table 10:** Connectivity associations between each of the bilateral subcortical structures with detected tractography and CH4. Displayed are slope (F-values) and p-values, adjusted for multiple comparisons using the Benjamini and Hochberg (1995) method.
